# Tri-μ-chlorido-bis­[(η^5^-penta­methyl­cyclo­penta­dien­yl)rhodium(III)] hexa­fluorido­phosphate from synchrotron radiation

**DOI:** 10.1107/S1600536813032480

**Published:** 2013-12-11

**Authors:** Lida Ezzedinloo, Sumi Shrestha, Mohan Bhadbhade, Stephen Colbran

**Affiliations:** a School of Chemistry, University of New South Wales, Sydney, NSW 2052, Australia; b Mark Wainwright Analytical Centre, University of New South Wales, Sydney, NSW 2052, Australia

## Abstract

In the title complex salt, [{(η^5^-C_5_Me_5_)Rh}_2_(μ-Cl)_3_]PF_6_, the dinuclear, single-charged cation is formed by the cojoining of two classic (η^5^-C_5_Me_5_)RhCl_3_ ‘piano-stool’ units by bridging of the three choride ligand ‘legs’. The crystal structure shows several close H⋯F contacts between the hexa­fluorido­phosphate counter-ions and the C_5_Me_5_ ligands.

## Related literature   

Complexes of the (η^5^-C_5_Me_5_)Rh^III^ group, modified by innumerable co-ligands, exhibit a diverse and very useful chemistry, particularly as homogeneous catalysts, see, for example: McSkimming *et al.* (2013 ▶); Brewster *et al.* (2013 ▶); Yu, Wan & Li (2013 ▶); Yu, Yu, Xiao, *et al.* (2013 ▶), Becerra *et al.* (2013 ▶); Gupta *et al.* (2013 ▶). The title complex salt, [{(η^5^-C_5_Me_5_)Rh}_2_(μ-Cl)_3_][PF_6_], is a commonly encountered impurity produced in reactions of the much-used Rh^III^ precursor [(η^5^-C_5_Me_5_)RhCl_2_]_2_ (Kang *et al.*, 1969 ▶; Booth *et al.*, 1969 ▶). [{(η^5^-C_5_Me_5_)Rh}_2_(μ-Cl)_3_][PF_6_] was first reported by Koelle (1990 ▶), and often (co-)crystallizes with or instead of the desired product of a reaction employing [(η^5^-C_5_Me_5_)RhCl_2_]_2_ and anion meta­thesis with a simple [PF_6_]^−^ salt. Several crystal structures of the [{(η^5^-C_5_Me_5_)Rh}_2_(μ-Cl)_3_]^+^ cation with other counter-ions have been reported, including salts with [PtCl_5_(CH_3_CONH_2_)]^−^ and [PtCl_6_]^2−^ (Umakoshi *et al.*, 1991 ▶), [{(C_6_F_5_)_2_Pd(μ-Cl)}_2_]^2−^ (Ara *et al.*, 2001 ▶), and BF_4_^−^ (Liu *et al.*, 2004 ▶) anions. 
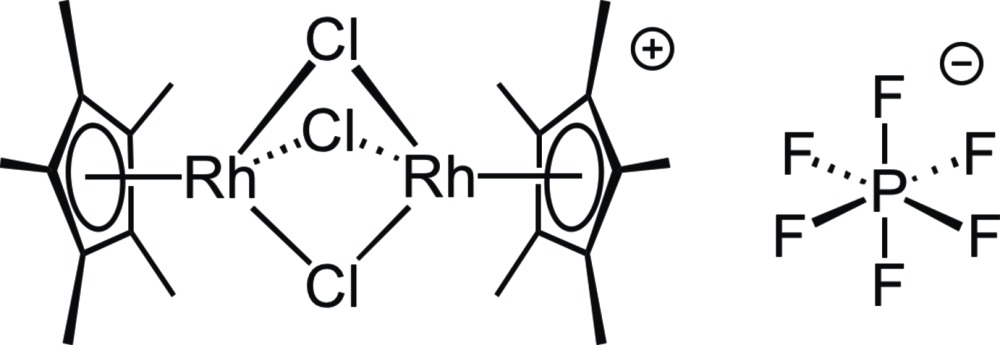


## Experimental   

### 

#### Crystal data  

[Rh_2_(C_10_H_15_)_2_Cl_3_]PF_6_
*M* *_r_* = 727.58
Triclinic, 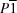

*a* = 8.0970 (16) Å
*b* = 12.604 (3) Å
*c* = 14.441 (3) Å
α = 64.28 (3)°
β = 82.42 (3)°
γ = 86.70 (3)°
*V* = 1316.1 (6) Å^3^
*Z* = 2
Synchrotron radiation
λ = 0.71073 Å
μ = 1.67 mm^−1^
*T* = 100 K
0.02 × 0.02 × 0.01 mm

#### Data collection  

3-BM1 Australian Synchrotron diffractometer
15787 measured reflections
4158 independent reflections
4064 reflections with *I* > 2σ(*I*)
*R*_int_ = 0.021

#### Refinement  

*R*[*F* ^2^ > 2σ(*F* ^2^)] = 0.025
*wR*(*F* ^2^) = 0.060
*S* = 1.09
4158 reflections
299 parameters
H-atom parameters constrained
Δρ_max_ = 1.03 e Å^−3^
Δρ_min_ = −0.57 e Å^−3^

### 

Data collection: *BLU-ICE* (McPhillips *et al.*, 2002 ▶); cell refinement: *XDS* (Kabsch, 1993 ▶); data reduction: *XDS*; program(s) used to solve structure: *SHELXS97* (Sheldrick, 2008 ▶) and *OLEX2* (Dolomanov *et al.*, 2009 ▶); program(s) used to refine structure: *SHELXL2013* (Sheldrick, 2008 ▶); molecular graphics: *CrystalMaker* (CrystalMaker, 2013 ▶); software used to prepare material for publication: *publCIF* (Westrip, 2010 ▶).

## Supplementary Material

Crystal structure: contains datablock(s) I. DOI: 10.1107/S1600536813032480/tk5276sup1.cif

Structure factors: contains datablock(s) I. DOI: 10.1107/S1600536813032480/tk5276Isup2.hkl

Additional supporting information:  crystallographic information; 3D view; checkCIF report

## Figures and Tables

**Table 1:** Hydrogen-bond geometry (Å, °)

*D*—H⋯*A*	*D*—H	H⋯*A*	*D*⋯*A*	*D*—H⋯*A*
C8*A*—H8*AB*⋯F3^i^	0.96	2.53	3.244 (5)	131
C10*A*—H10*A*⋯F1^ii^	0.96	2.43	3.328 (4)	156
C6*B*—H6*BB*⋯F1	0.96	2.49	3.152 (5)	126

Symmetry codes: (i) 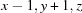; (ii) 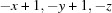.

**Fig. 1.**
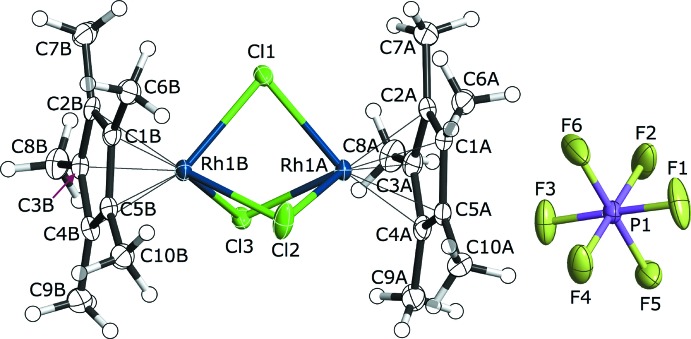
View of [{(η5-C5Me5)Rh}2(µ-Cl)3][PF6] showing 50% displacement ellipsoids at 100 K and the atom labelling scheme.

**Fig. 2.**
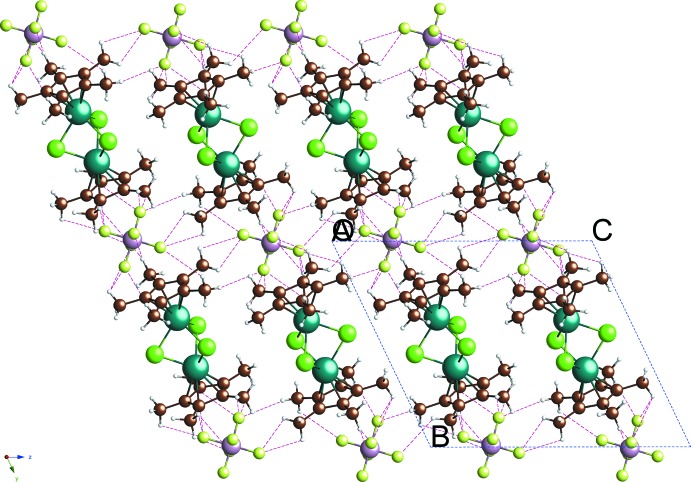
View of the crystal structure of [{(η5-C5Me5)Rh}2(µ-Cl)3][PF6] illustrating the segregation of cations and anions into interleaved layers packed parallel to the b-axis and with the shorter H···F cation-anion contacts highlighted by dashed lines.

## supplementary crystallographic information

### 2.1. Synthesis and crystallization

Precursor [(η^5^-C_5_Me_5_)RhCl_2_]_2_ (Kang *et al.*, 1969 and Booth *et al.*, 1969) was dissolved in methanol and treated with a saturated aqueous solution of K[PF_6_]. The orange precipitate was collected, and was recrystallized from acetone–methanol overnight at 4 °C to afford the thin orange crystalline needles of [{(η^5^-C_5_Me_5_)Rh}_2_(µ-Cl)_3_][PF_6_] that were used for this X-ray crystal structure determination.

### 2.2. Refinement

Crystal data, data collection and structure refinement details are summarized in Table 1. The H atoms were geometrically placed (C—H = 0.96 Å) and refined as riding with *U_iso_*(H) = 1.25*U_eq_*(C).

### 3. Results and discussion

Fig. 1 presents a view of the [{(η^5^-C_5_Me_5_)Rh}_2_(µ-Cl)_3_]^+^ cation and the [PF_6_]^-^ anion. In the [{(η^5^-C_5_Me_5_)Rh}_2_(µ-Cl)_3_]^+^ cation, the two independent Rh^III^ ions each exhibit pseudo-o­cta­hedral geometry with a C_5_Me_5_ group occupying three coordination sites and three bridging chlorido ligands the other three. The Rh–centroid (C5) distances are 2.12 and 2.15 Å and the mean Rh–Cl(bridge) bond length is 2.455 Å. The intra­molecular Rh–Rh distance is 3.21 Å, consistent with the absence of a metal–metal bond as predicted by the 18-electron rule, and the mean Rh–Cl–Rh angle is noticeably acute at 82°.

Fig. 2 presents a 'ball-and-stick view' of the packing of the ions in the crystal structure. The hexafluoridophosphate counterions lie in layers parallel to the *b*-axis that inter­leave between layers of the cations. There are close C—H···F contacts, Table 1, between the [PF_6_]^-^ ions and the methyl groups of the [{(η^5^-C_5_Me_5_)Rh}_2_(µ-Cl)_3_]^+^ cations.

### Crystal data

**Table:**

[Rh_2_(C_10_H_15_)_2_Cl_3_]PF_6_	*Z* = 2
*M**_r_* = 727.58	*F*(000) = 720
Triclinic, *P*1	*D*_x_ = 1.836 Mg m^−^^3^
*a* = 8.0970 (16) Å	Synchrotron radiation, λ = 0.71073 Å
*b* = 12.604 (3) Å	Cell parameters from 9980 reflections
*c* = 14.441 (3) Å	θ = 2.5–22.5°
α = 64.28 (3)°	µ = 1.67 mm^−^^1^
β = 82.42 (3)°	*T* = 100 K
γ = 86.70 (3)°	Plate, yellow
*V* = 1316.1 (6) Å^3^	0.02 × 0.02 × 0.01 mm

### Data collection

**Table:**

3-BM1 Australian Synchrotron diffractometer	4064 reflections with *I* > 2σ(*I*)
Radiation source: Synchrotron BM	*R*_int_ = 0.021
Si<111> monochromator	θ_max_ = 25.0°, θ_min_ = 1.8°
Phi Scan scans	*h* = −9→9
15787 measured reflections	*k* = −14→14
4158 independent reflections	*l* = −17→17

### Refinement

**Table:**

Refinement on *F*^2^	0 restraints
Least-squares matrix: full	Hydrogen site location: inferred from neighbouring sites
*R*[*F*^2^ > 2σ(*F*^2^)] = 0.025	H-atom parameters constrained
*wR*(*F*^2^) = 0.060	*w* = 1/[σ^2^(*F*_o_^2^) + (0.018*P*)^2^ + 3.0381*P*] where *P* = (*F*_o_^2^ + 2*F*_c_^2^)/3
*S* = 1.09	(Δ/σ)_max_ = 0.001
4158 reflections	Δρ_max_ = 1.03 e Å^−^^3^
299 parameters	Δρ_min_ = −0.57 e Å^−^^3^

### Special details

**Table:**

Geometry. All e.s.d.'s (except the e.s.d. in the dihedral angle between two l.s. planes) are estimated using the full covariance matrix. The cell e.s.d.'s are taken into account individually in the estimation of e.s.d.'s in distances, angles and torsion angles; correlations between e.s.d.'s in cell parameters are only used when they are defined by crystal symmetry. An approximate (isotropic) treatment of cell e.s.d.'s is used for estimating e.s.d.'s involving l.s. planes.
Refinement. Refinement of *F^2^* is against ALL reflections. The weighted *R*-factor w*R* and goodness of fit *S* are based on *F^2^*; conventional *R*-factors *R* are based on *F*, with *F* set to zero for negative *F^2^*. The threshold expression of *F^2^* > σ(*F^2^*) is used only for calculating *R*-factors (gt) *etc*. and is not relevant to the choice of reflections for refinement. *R*-factors based on *F^2^* are statistically about twice as large as those based on *F*, and *R*-factors based on ALL data will be even larger.

### Fractional atomic coordinates and isotropic or equivalent isotropic displacement parameters (Å^2^)

**Table:**

	*x*	*y*	*z*	*U*_iso_*/*U*_eq_	
Rh1A	0.10004 (3)	0.61463 (2)	0.24284 (2)	0.01511 (8)	
C1A	0.1288 (4)	0.7995 (2)	0.1858 (2)	0.0193 (6)	
C2A	0.0856 (4)	0.7501 (2)	0.2954 (2)	0.0204 (6)	
C3A	−0.0722 (4)	0.6920 (2)	0.3203 (3)	0.0216 (6)	
C4A	−0.1288 (4)	0.7091 (2)	0.2238 (2)	0.0205 (6)	
C5A	−0.0069 (4)	0.7767 (2)	0.1414 (2)	0.0195 (6)	
C6A	0.2816 (4)	0.8692 (3)	0.1264 (3)	0.0270 (7)	
H6AA	0.3726	0.8414	0.1675	0.041*	
H6AB	0.2616	0.9509	0.1102	0.041*	
H6AC	0.3089	0.8602	0.0635	0.041*	
C7A	0.1867 (4)	0.7580 (3)	0.3717 (3)	0.0290 (7)	
H7AA	0.1621	0.6918	0.4376	0.044*	
H7AB	0.1599	0.8296	0.3786	0.044*	
H7AC	0.3030	0.7576	0.3478	0.044*	
C8A	−0.1641 (4)	0.6277 (3)	0.4248 (3)	0.0288 (7)	
H8AA	−0.2167	0.5596	0.4283	0.043*	
H8AB	−0.2473	0.6784	0.4380	0.043*	
H8AC	−0.0877	0.6035	0.4758	0.043*	
C9A	−0.2884 (4)	0.6647 (3)	0.2138 (3)	0.0283 (7)	
H9AA	−0.2774	0.6544	0.1512	0.043*	
H9AB	−0.3754	0.7205	0.2116	0.043*	
H9AC	−0.3155	0.5906	0.2721	0.043*	
C10A	−0.0175 (4)	0.8173 (3)	0.0292 (3)	0.0258 (7)	
H10A	0.0927	0.8243	−0.0069	0.039*	
H10B	−0.0720	0.8925	0.0025	0.039*	
H10C	−0.0800	0.7613	0.0193	0.039*	
Cl1	0.34572 (8)	0.51534 (6)	0.32353 (6)	0.02165 (16)	
Cl2	0.21608 (12)	0.55011 (7)	0.11061 (6)	0.0337 (2)	
Cl3	−0.00889 (8)	0.40972 (6)	0.32708 (6)	0.02133 (16)	
Rh1B	0.27583 (3)	0.37186 (2)	0.26318 (2)	0.01627 (8)	
C1B	0.5043 (4)	0.2970 (2)	0.2305 (2)	0.0200 (6)	
C2B	0.4541 (4)	0.2404 (2)	0.3399 (2)	0.0208 (6)	
C3B	0.2944 (4)	0.1876 (2)	0.3568 (3)	0.0230 (6)	
C4B	0.2479 (4)	0.2083 (2)	0.2568 (3)	0.0226 (6)	
C5B	0.3795 (4)	0.2738 (2)	0.1797 (2)	0.0212 (6)	
C6B	0.6637 (4)	0.3625 (3)	0.1790 (3)	0.0250 (7)	
H6BA	0.6465	0.4236	0.1122	0.038*	
H6BB	0.7476	0.3091	0.1710	0.038*	
H6BC	0.6995	0.3968	0.2208	0.038*	
C7B	0.5525 (4)	0.2373 (3)	0.4214 (3)	0.0254 (7)	
H7BA	0.6112	0.3103	0.3967	0.038*	
H7BB	0.6309	0.1735	0.4373	0.038*	
H7BC	0.4785	0.2260	0.4826	0.038*	
C8B	0.1955 (4)	0.1180 (3)	0.4585 (3)	0.0305 (7)	
H8BA	0.2270	0.1400	0.5095	0.046*	
H8BB	0.2166	0.0356	0.4791	0.046*	
H8BC	0.0790	0.1336	0.4523	0.046*	
C9B	0.0951 (4)	0.1628 (3)	0.2385 (3)	0.0310 (7)	
H9BA	0.0070	0.1553	0.2925	0.046*	
H9BB	0.1183	0.0873	0.2386	0.046*	
H9BC	0.0618	0.2167	0.1728	0.046*	
C10B	0.3836 (4)	0.3111 (3)	0.0667 (3)	0.0273 (7)	
H10D	0.2756	0.3394	0.0460	0.041*	
H10E	0.4137	0.2451	0.0513	0.041*	
H10F	0.4642	0.3727	0.0297	0.041*	
P1	0.65562 (9)	−0.00832 (6)	0.23670 (6)	0.02081 (17)	
F1	0.6989 (3)	0.12409 (17)	0.1566 (2)	0.0543 (7)	
F2	0.6292 (4)	0.0304 (2)	0.32864 (19)	0.0580 (8)	
F3	0.6095 (3)	−0.14070 (16)	0.31547 (17)	0.0444 (6)	
F4	0.6793 (3)	−0.04801 (18)	0.14494 (16)	0.0435 (5)	
F5	0.4642 (2)	0.01935 (18)	0.2209 (2)	0.0425 (5)	
F6	0.8456 (3)	−0.0364 (2)	0.2528 (3)	0.0699 (9)	

### Atomic displacement parameters (Å^2^)

**Table:**

	*U*^11^	*U*^22^	*U*^33^	*U*^12^	*U*^13^	*U*^23^
Rh1A	0.01543 (12)	0.01295 (12)	0.01698 (14)	0.00227 (8)	−0.00149 (9)	−0.00688 (9)
C1A	0.0213 (14)	0.0129 (13)	0.0248 (17)	0.0034 (11)	−0.0064 (13)	−0.0084 (11)
C2A	0.0217 (14)	0.0168 (13)	0.0266 (17)	0.0056 (11)	−0.0052 (13)	−0.0129 (12)
C3A	0.0227 (14)	0.0176 (14)	0.0253 (18)	0.0061 (11)	−0.0002 (13)	−0.0113 (12)
C4A	0.0182 (14)	0.0162 (13)	0.0274 (18)	0.0059 (11)	−0.0025 (13)	−0.0105 (12)
C5A	0.0202 (14)	0.0122 (12)	0.0249 (17)	0.0029 (10)	−0.0050 (13)	−0.0067 (11)
C6A	0.0234 (15)	0.0205 (15)	0.0341 (19)	−0.0017 (12)	−0.0068 (14)	−0.0078 (13)
C7A	0.0357 (17)	0.0268 (16)	0.0307 (19)	0.0050 (13)	−0.0125 (15)	−0.0163 (14)
C8A	0.0305 (16)	0.0269 (16)	0.0252 (19)	0.0057 (13)	0.0045 (15)	−0.0106 (14)
C9A	0.0197 (15)	0.0236 (15)	0.043 (2)	0.0006 (12)	−0.0042 (14)	−0.0151 (14)
C10A	0.0277 (16)	0.0245 (15)	0.0255 (18)	0.0022 (12)	−0.0082 (14)	−0.0098 (13)
Cl1	0.0140 (3)	0.0187 (3)	0.0367 (4)	0.0013 (2)	−0.0064 (3)	−0.0153 (3)
Cl2	0.0535 (5)	0.0247 (4)	0.0181 (4)	0.0177 (3)	−0.0013 (4)	−0.0077 (3)
Cl3	0.0147 (3)	0.0172 (3)	0.0337 (4)	0.0003 (2)	−0.0044 (3)	−0.0121 (3)
Rh1B	0.01803 (12)	0.01362 (12)	0.01822 (14)	0.00375 (8)	−0.00374 (9)	−0.00785 (9)
C1B	0.0216 (14)	0.0185 (13)	0.0239 (17)	0.0078 (11)	−0.0064 (13)	−0.0127 (12)
C2B	0.0217 (14)	0.0150 (13)	0.0265 (18)	0.0089 (11)	−0.0085 (13)	−0.0093 (12)
C3B	0.0273 (15)	0.0141 (13)	0.0277 (18)	0.0060 (11)	−0.0076 (14)	−0.0086 (12)
C4B	0.0249 (15)	0.0163 (13)	0.0303 (18)	0.0043 (11)	−0.0066 (14)	−0.0131 (12)
C5B	0.0227 (14)	0.0179 (14)	0.0278 (18)	0.0072 (11)	−0.0071 (13)	−0.0141 (12)
C6B	0.0224 (15)	0.0253 (15)	0.0315 (19)	0.0044 (12)	−0.0046 (14)	−0.0162 (14)
C7B	0.0280 (16)	0.0258 (15)	0.0238 (18)	0.0053 (12)	−0.0089 (14)	−0.0110 (13)
C8B	0.0335 (17)	0.0220 (15)	0.0293 (19)	−0.0002 (13)	−0.0017 (15)	−0.0054 (13)
C9B	0.0277 (16)	0.0284 (16)	0.043 (2)	0.0001 (13)	−0.0100 (16)	−0.0198 (15)
C10B	0.0297 (16)	0.0304 (16)	0.0290 (19)	0.0074 (13)	−0.0097 (15)	−0.0187 (14)
P1	0.0211 (4)	0.0157 (4)	0.0277 (5)	0.0025 (3)	−0.0044 (3)	−0.0112 (3)
F1	0.0556 (14)	0.0169 (10)	0.0746 (19)	−0.0048 (9)	0.0307 (13)	−0.0151 (10)
F2	0.100 (2)	0.0476 (13)	0.0488 (15)	0.0435 (14)	−0.0434 (15)	−0.0372 (12)
F3	0.0634 (14)	0.0196 (9)	0.0375 (13)	0.0082 (9)	0.0102 (11)	−0.0058 (9)
F4	0.0713 (15)	0.0319 (11)	0.0294 (12)	0.0141 (10)	−0.0040 (11)	−0.0174 (9)
F5	0.0265 (10)	0.0384 (11)	0.0757 (17)	0.0103 (8)	−0.0171 (11)	−0.0348 (11)
F6	0.0297 (11)	0.0550 (14)	0.163 (3)	0.0194 (10)	−0.0407 (15)	−0.0760 (18)

### Geometric parameters (Å, º)

**Table:**

Rh1A—C1A	2.123 (3)	Rh1B—C1B	2.119 (3)
Rh1A—C4A	2.126 (3)	Rh1B—C4B	2.129 (3)
Rh1A—C3A	2.127 (3)	Rh1B—C3B	2.129 (3)
Rh1A—C2A	2.140 (3)	Rh1B—C5B	2.142 (3)
Rh1A—C5A	2.145 (3)	Rh1B—C2B	2.147 (3)
Rh1A—Cl1	2.4372 (11)	C1B—C2B	1.434 (5)
Rh1A—Cl2	2.4448 (10)	C1B—C5B	1.435 (4)
Rh1A—Cl3	2.4860 (11)	C1B—C6B	1.496 (4)
C1A—C2A	1.428 (5)	C2B—C3B	1.433 (4)
C1A—C5A	1.443 (4)	C2B—C7B	1.491 (4)
C1A—C6A	1.493 (4)	C3B—C4B	1.449 (4)
C2A—C3A	1.434 (4)	C3B—C8B	1.490 (5)
C2A—C7A	1.496 (4)	C4B—C5B	1.430 (5)
C3A—C4A	1.447 (4)	C4B—C9B	1.492 (4)
C3A—C8A	1.483 (5)	C5B—C10B	1.486 (5)
C4A—C5A	1.423 (4)	C6B—H6BA	0.9600
C4A—C9A	1.485 (4)	C6B—H6BB	0.9600
C5A—C10A	1.485 (4)	C6B—H6BC	0.9600
C6A—H6AA	0.9600	C7B—H7BA	0.9600
C6A—H6AB	0.9600	C7B—H7BB	0.9600
C6A—H6AC	0.9600	C7B—H7BC	0.9600
C7A—H7AA	0.9600	C8B—H8BA	0.9600
C7A—H7AB	0.9600	C8B—H8BB	0.9600
C7A—H7AC	0.9600	C8B—H8BC	0.9600
C8A—H8AA	0.9600	C9B—H9BA	0.9600
C8A—H8AB	0.9600	C9B—H9BB	0.9600
C8A—H8AC	0.9600	C9B—H9BC	0.9600
C9A—H9AA	0.9600	C10B—H10D	0.9600
C9A—H9AB	0.9600	C10B—H10E	0.9600
C9A—H9AC	0.9600	C10B—H10F	0.9600
C10A—H10A	0.9600	P1—F6	1.583 (2)
C10A—H10B	0.9600	P1—F2	1.589 (2)
C10A—H10C	0.9600	P1—F3	1.590 (2)
Cl1—Rh1B	2.4426 (9)	P1—F1	1.591 (2)
Cl2—Rh1B	2.4485 (13)	P1—F5	1.593 (2)
Cl3—Rh1B	2.4675 (10)	P1—F4	1.594 (2)
			
C1A—Rh1A—C4A	66.16 (11)	C1B—Rh1B—C2B	39.27 (12)
C1A—Rh1A—C3A	66.24 (12)	C4B—Rh1B—C2B	65.99 (11)
C4A—Rh1A—C3A	39.78 (12)	C3B—Rh1B—C2B	39.15 (12)
C1A—Rh1A—C2A	39.13 (12)	C5B—Rh1B—C2B	65.51 (11)
C4A—Rh1A—C2A	65.91 (11)	C1B—Rh1B—Cl1	106.37 (8)
C3A—Rh1A—C2A	39.26 (12)	C4B—Rh1B—Cl1	160.83 (9)
C1A—Rh1A—C5A	39.53 (11)	C3B—Rh1B—Cl1	121.24 (9)
C4A—Rh1A—C5A	38.91 (12)	C5B—Rh1B—Cl1	142.82 (8)
C3A—Rh1A—C5A	65.94 (12)	C2B—Rh1B—Cl1	96.84 (8)
C2A—Rh1A—C5A	65.55 (11)	C1B—Rh1B—Cl2	109.99 (9)
C1A—Rh1A—Cl1	109.38 (8)	C4B—Rh1B—Cl2	116.63 (9)
C4A—Rh1A—Cl1	158.78 (9)	C3B—Rh1B—Cl2	156.37 (9)
C3A—Rh1A—Cl1	119.00 (9)	C5B—Rh1B—Cl2	95.96 (9)
C2A—Rh1A—Cl1	97.15 (8)	C2B—Rh1B—Cl2	147.91 (9)
C5A—Rh1A—Cl1	146.87 (8)	Cl1—Rh1B—Cl2	82.39 (3)
C1A—Rh1A—Cl2	110.16 (9)	C1B—Rh1B—Cl3	166.37 (8)
C4A—Rh1A—Cl2	118.62 (9)	C4B—Rh1B—Cl3	102.35 (9)
C3A—Rh1A—Cl2	158.36 (9)	C3B—Rh1B—Cl3	100.28 (10)
C2A—Rh1A—Cl2	147.40 (9)	C5B—Rh1B—Cl3	134.81 (8)
C5A—Rh1A—Cl2	97.28 (9)	C2B—Rh1B—Cl3	130.39 (9)
Cl1—Rh1A—Cl2	82.58 (3)	Cl1—Rh1B—Cl3	81.93 (3)
C1A—Rh1A—Cl3	164.71 (8)	Cl2—Rh1B—Cl3	81.46 (5)
C4A—Rh1A—Cl3	99.66 (9)	C2B—C1B—C5B	108.0 (3)
C3A—Rh1A—Cl3	99.37 (9)	C2B—C1B—C6B	125.8 (3)
C2A—Rh1A—Cl3	131.24 (9)	C5B—C1B—C6B	126.1 (3)
C5A—Rh1A—Cl3	131.26 (8)	C2B—C1B—Rh1B	71.41 (16)
Cl1—Rh1A—Cl3	81.65 (4)	C5B—C1B—Rh1B	71.21 (16)
Cl2—Rh1A—Cl3	81.16 (4)	C6B—C1B—Rh1B	125.8 (2)
C2A—C1A—C5A	107.8 (3)	C3B—C2B—C1B	108.2 (3)
C2A—C1A—C6A	126.4 (3)	C3B—C2B—C7B	126.3 (3)
C5A—C1A—C6A	125.7 (3)	C1B—C2B—C7B	125.6 (3)
C2A—C1A—Rh1A	71.08 (16)	C3B—C2B—Rh1B	69.76 (16)
C5A—C1A—Rh1A	71.05 (15)	C1B—C2B—Rh1B	69.32 (15)
C6A—C1A—Rh1A	126.3 (2)	C7B—C2B—Rh1B	126.7 (2)
C1A—C2A—C3A	108.5 (3)	C2B—C3B—C4B	107.8 (3)
C1A—C2A—C7A	126.0 (3)	C2B—C3B—C8B	126.9 (3)
C3A—C2A—C7A	125.5 (3)	C4B—C3B—C8B	125.2 (3)
C1A—C2A—Rh1A	69.79 (16)	C2B—C3B—Rh1B	71.09 (16)
C3A—C2A—Rh1A	69.86 (16)	C4B—C3B—Rh1B	70.09 (16)
C7A—C2A—Rh1A	126.5 (2)	C8B—C3B—Rh1B	126.6 (2)
C2A—C3A—C4A	107.4 (3)	C5B—C4B—C3B	107.7 (3)
C2A—C3A—C8A	127.1 (3)	C5B—C4B—C9B	126.3 (3)
C4A—C3A—C8A	125.5 (3)	C3B—C4B—C9B	125.9 (3)
C2A—C3A—Rh1A	70.88 (17)	C5B—C4B—Rh1B	70.96 (16)
C4A—C3A—Rh1A	70.09 (17)	C3B—C4B—Rh1B	70.12 (16)
C8A—C3A—Rh1A	125.2 (2)	C9B—C4B—Rh1B	127.2 (2)
C5A—C4A—C3A	108.2 (3)	C4B—C5B—C1B	108.3 (3)
C5A—C4A—C9A	126.5 (3)	C4B—C5B—C10B	125.2 (3)
C3A—C4A—C9A	125.3 (3)	C1B—C5B—C10B	126.5 (3)
C5A—C4A—Rh1A	71.25 (16)	C4B—C5B—Rh1B	69.91 (17)
C3A—C4A—Rh1A	70.13 (16)	C1B—C5B—Rh1B	69.44 (16)
C9A—C4A—Rh1A	125.1 (2)	C10B—C5B—Rh1B	126.2 (2)
C4A—C5A—C1A	108.0 (3)	C1B—C6B—H6BA	109.5
C4A—C5A—C10A	126.1 (3)	C1B—C6B—H6BB	109.5
C1A—C5A—C10A	125.9 (3)	H6BA—C6B—H6BB	109.5
C4A—C5A—Rh1A	69.83 (16)	C1B—C6B—H6BC	109.5
C1A—C5A—Rh1A	69.42 (15)	H6BA—C6B—H6BC	109.5
C10A—C5A—Rh1A	126.6 (2)	H6BB—C6B—H6BC	109.5
C1A—C6A—H6AA	109.5	C2B—C7B—H7BA	109.5
C1A—C6A—H6AB	109.5	C2B—C7B—H7BB	109.5
H6AA—C6A—H6AB	109.5	H7BA—C7B—H7BB	109.5
C1A—C6A—H6AC	109.5	C2B—C7B—H7BC	109.5
H6AA—C6A—H6AC	109.5	H7BA—C7B—H7BC	109.5
H6AB—C6A—H6AC	109.5	H7BB—C7B—H7BC	109.5
C2A—C7A—H7AA	109.5	C3B—C8B—H8BA	109.5
C2A—C7A—H7AB	109.5	C3B—C8B—H8BB	109.5
H7AA—C7A—H7AB	109.5	H8BA—C8B—H8BB	109.5
C2A—C7A—H7AC	109.5	C3B—C8B—H8BC	109.5
H7AA—C7A—H7AC	109.5	H8BA—C8B—H8BC	109.5
H7AB—C7A—H7AC	109.5	H8BB—C8B—H8BC	109.5
C3A—C8A—H8AA	109.5	C4B—C9B—H9BA	109.5
C3A—C8A—H8AB	109.5	C4B—C9B—H9BB	109.5
H8AA—C8A—H8AB	109.5	H9BA—C9B—H9BB	109.5
C3A—C8A—H8AC	109.5	C4B—C9B—H9BC	109.5
H8AA—C8A—H8AC	109.5	H9BA—C9B—H9BC	109.5
H8AB—C8A—H8AC	109.5	H9BB—C9B—H9BC	109.5
C4A—C9A—H9AA	109.5	C5B—C10B—H10D	109.5
C4A—C9A—H9AB	109.5	C5B—C10B—H10E	109.5
H9AA—C9A—H9AB	109.5	H10D—C10B—H10E	109.5
C4A—C9A—H9AC	109.5	C5B—C10B—H10F	109.5
H9AA—C9A—H9AC	109.5	H10D—C10B—H10F	109.5
H9AB—C9A—H9AC	109.5	H10E—C10B—H10F	109.5
C5A—C10A—H10A	109.5	F6—P1—F2	90.85 (15)
C5A—C10A—H10B	109.5	F6—P1—F3	89.65 (15)
H10A—C10A—H10B	109.5	F2—P1—F3	90.74 (14)
C5A—C10A—H10C	109.5	F6—P1—F1	91.30 (15)
H10A—C10A—H10C	109.5	F2—P1—F1	89.88 (15)
H10B—C10A—H10C	109.5	F3—P1—F1	178.86 (16)
Rh1A—Cl1—Rh1B	82.26 (3)	F6—P1—F5	179.7 (2)
Rh1A—Cl2—Rh1B	81.99 (3)	F2—P1—F5	89.10 (14)
Rh1B—Cl3—Rh1A	80.78 (4)	F3—P1—F5	90.11 (13)
C1B—Rh1B—C4B	66.30 (12)	F1—P1—F5	88.95 (13)
C1B—Rh1B—C3B	66.24 (12)	F6—P1—F4	89.88 (15)
C4B—Rh1B—C3B	39.79 (12)	F2—P1—F4	179.11 (16)
C1B—Rh1B—C5B	39.35 (11)	F3—P1—F4	88.76 (12)
C4B—Rh1B—C5B	39.13 (12)	F1—P1—F4	90.60 (13)
C3B—Rh1B—C5B	65.93 (12)	F5—P1—F4	90.17 (13)

### Hydrogen-bond geometry (Å, º)

**Table:**

*D*—H···*A*	*D*—H	H···*A*	*D*···*A*	*D*—H···*A*
C8*A*—H8*AB*···F3^i^	0.96	2.53	3.244 (5)	131
C10*A*—H10*A*···F1^ii^	0.96	2.43	3.328 (4)	156
C6*B*—H6*BB*···F1	0.96	2.49	3.152 (5)	126

Symmetry codes: (i) *x*−1, *y*+1, *z*; (ii) −*x*+1, −*y*+1, −*z*.
